# Supplementary material for: The Mitochondrial PHB2/OMA1/DELE1 Pathway Cooperates with Endoplasmic Reticulum Stress to Facilitate the Response to Chemotherapeutics in Ovarian Cancer
Source: Int J Mol Sci. 2022 Jan 25;23(3):1320. doi: 10.3390/ijms23031320 (PMC8835964; doi:10.3390/ijms23031320)
Supplement: Supplementary file 1 [file ijms-23-01320-s001.zip › ijms-1536468-supplementary.pdf]

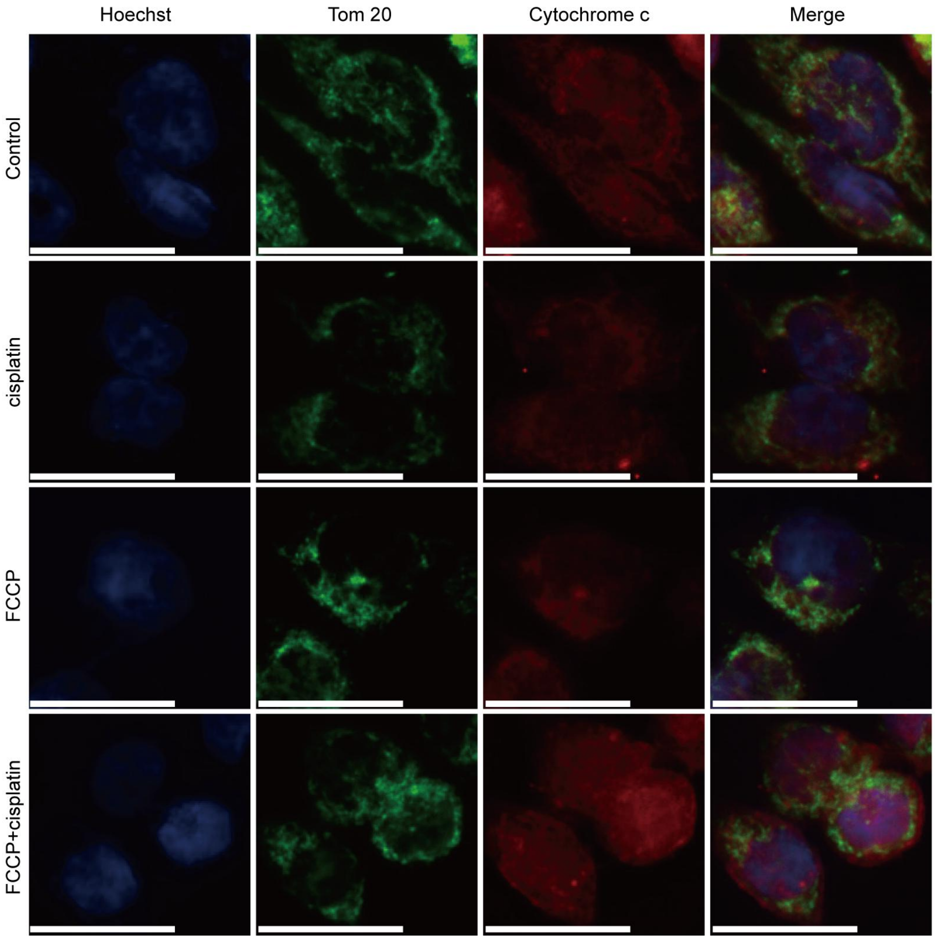

Figure S1. FCCP combined with cisplatin induced cytochrome c release. A2780 cells were treated with FCCP and/or cisplatin for 12 h. Colocalization of cytochrome c with Tom20 in A2780 cells was measured by immunofluorescence staining (scale bar, 30 μm).
